# Synthesis, Characterization, and Biological Evaluation of Unimetallic and Heterobimetallic Complexes of Bivalent Copper

**DOI:** 10.1155/2018/2467463

**Published:** 2018-04-12

**Authors:** Anshul Singh, Ashu Chaudhary

**Affiliations:** Department of Chemistry, Kurukshetra University, Kurukshetra 136 119, India

## Abstract

We present an inclusive characterization of the unimetallic and heterobimetallic complexes of copper synthesized using CuCl_2_ and diamine (4-fluoro 1,2-phenylenediamine) resulting in monometallic complex which further undergoes treatment with organometallic dichlorides of group 4 and 14 in 1 : 2 molar ratio resulting in heterobimetallic complexes. These complexes thoroughly characterized using various physical, analytical, and spectroscopic techniques indicate square planar and distorted octahedral geometry for the synthesized unimetallic and heterobimetallic complexes, respectively. These complexes were evaluated for their antimicrobial efficacy against various bacterial and fungal strains while hepatoprotective activity was also examined in male albino rats.

## 1. Introduction

The advances in the field of bioinorganic chemistry have augmented the interest in heterobimetallic complexes since it has been recognized that many of these complexes may serve as biologically significant species [1]. The biomedical inorganic chemistry has been a captivating research area due to extensive applications of inorganic pharmaceuticals in innumerable clinical therapy and diagnosis [2]. Transition metal complexes have received much attention because of their biological activities, including antiviral [3], anticarcinogenic [4], antifertility [5], antibacterial [6], and antifungal activities [7].

Among the transition metals, copper has been the most studied metal ion. It is one of the trace elements essential for human life, vital for the function of enzymes, protein and DNA synthesis, and also in the regulation of intracellular redox potential. Ceruloplasmin, the major copper-carrying protein in the blood is bound to almost all the copper prevailing in the serum of living organisms. There is a reduction of copper from oxidation state +2 to +1 by metalloreductases at the cell surface, before uptake into the cell [8]. The chemistry of copper has been ruled essentially by copper(II) compounds, chiefly due to the difficulty arising in stabilizing copper(I) species. Due to the ineffable biological significance, an enormous amount of copper(II) complexes have been synthesized and explored for their biological activities [9–14].

The emphasis has been laid down on the design of heterobimetallic complexes based on the inference that combining two different metal centers in the same molecule results into the substantial modification of reactivity over that of their monometallic species. The provocative properties of heterobimetallic complexes ascend from the possible “synergistic” effect of two different metal ions held together in close proximity [15]. These annotations have emboldened chemists to synthesize new heterobimetallic complexes comprising two different metal centers with an impression being that such a system will be more efficacious than the unimetallic species involving the individual metal centers for numerous fundamental reactions [16]. Considering the significant role of Cu(II) played in human beings, in addition to its proficient biological activity, we have synthesized and characterized the mononuclear and heterobimetallic Cu(II) complexes with organometallic dichlorides of group 4 and 14 in the presence of 4-fluoro 1,2-phenylenediamine in the current study. Besides the characterization of complexes by various physicochemical techniques, their examination against bacterial strains, namely, *Escherichia coli* (MTCC1687) and *Staphylococcus aureus* (MTCC96) and some fungal strains, namely, *Fusarium oxysporum* and *Aspergillus niger* was carried out with antibiotic streptomycin and Bavistin used as standards.

Apart from antimicrobial activity, hepatoprotectivity of the complexes was also evaluated using male albino rats. The imperative functions in regulating and maintaining homeostasis of the body are performed by the liver, which is also known for its vital role in fat metabolism, bile secretion, storage of vitamins, and detoxification of endogenous and exogenous substances. Therefore, to remain healthy is crucial to maintain a healthy liver [17]. Hepatotoxicity entails chemical-driven liver damage. The considerable cause for the occurrence of iatrogenic diseases is drug-induced hepatotoxicity, and hence, there is an acute necessity for developing reliable hepatoprotective drugs contributing towards the current medical practice. Hence, the synthesized heterobimetallic complexes of copper were inspected for their hepatoprotective activity in male albino rats, and the results obtained were quite encouraging.

## 2. Experimental

### 2.1. Materials

All the chemicals used were of AnalaR grade. CuCl_2_, 4-fluoro 1,2-phenylenediamine, and organometallic dichlorides were purchased from Sigma-Aldrich and used as obtained. The solvents used were purchased from HiMedia and were distilled and dried before use.

### 2.2. Physical Measurements and Analytical Methods

The IR spectra (4000–200 cm^−1^) were recorded on a Nicolet Magna FTIR-550 spectrophotometer in the form of KBr pellets while the far infrared spectra of the complexes were recorded on the same spectrophotometer in Nujol mulls using CsI cell. Conductivity measurements of the compounds were measured in 10^−3^ M DMF solution on a Century Digital conductivity meter model CC601. Electronic spectra were recorded on a UV-visible spectrophotometer 752/752N. X-ray powder diffraction spectra of the compound were obtained on PANalytical X'pert Pro 3040 Almelo, while EPR spectra of the complexes were recorded on a Varian E-4 EPR spectrometer. Molecular weights were determined by the Rast Camphor method. Metal contents were estimated gravimetrically. Nitrogen and chlorine were estimated by the Kjeldahl's method and the Volhard's method, respectively.

### 2.3. Synthesis of Monometallic Complex [Cu(C_6_H_7_N_2_F)_2_]Cl_2_

The monometallic complex was prepared by dissolving anhydrous CuCl_2_ (1.34 g, 0.01 mol) in hot methanol in a 100 mL round-bottomed flask to which the required amount of diamine, that is, 4-fluoro 1,2-phenylenediamine (2.52 g, 0.02 mol) was added drop wise in 1 : 2 stoichiometric ratio under constant stirring. The product thus obtained was washed with methanol and recrystallized from a 1 : 1 solution of benzene and methanol and then dried in vacuo.

### 2.4. Synthesis of Heterobimetallic Complex [Cu(C_6_H_5_N_2_F)_2_Sn_2_(Ph)_4_Cl_2_]

This complex was obtained by mixing a methanolic solution of monometallic complex [Cu(C_6_H_7_N_2_F)_2_]Cl_2_ (3.86 g, 0.01 mol) to a methanolic solution of Ph_2_SnCl_2_ (6.88 g, 0.02 mol) in 1 : 2 stoichiometric proportions under constant stirring. The mixture resulted into a solid product, obtained after keeping the reaction mixture overnight at room temperature. The colored product thus attained was washed with methanol and then dried in vacuo. All the other complexes were synthesized using the same procedure by replacing Ph_2_SnCl_2_ with (CH_3_)_2_SnCl_2_, Ph_2_SiCl_2_, (Cp)_2_TiCl_2,_ and (Cp)_2_ZrCl_2,_ respectively.

**Scheme 1:**
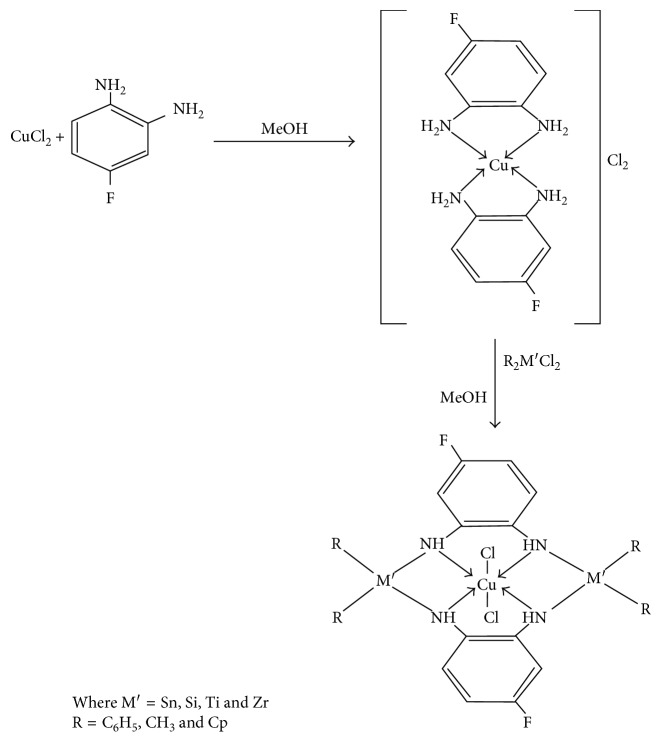


The monometallic and heterobimetallic complexes reported in the current work were synthesized as outlined in Scheme 1.

### 2.5. Biological Assay

#### 2.5.1. Test Microorganisms

Unimetallic complex of Cu(II) and its corresponding heterobimetallic complexes have been examined for their fungicidal and bactericidal efficacy. The bacterial and fungal species used for evaluating antimicrobial activity of the synthesized complexes were *Escherichia coli*, *Staphylococcusaureus*, *Fusarium oxysporum*, and *Aspergillus niger*.

*(1) Antibacterial Activity*. The synthesized complexes were appraised for their antibacterial activity against the bacterial strains *Escherichia coli* (MTCC1687) and *Staphylococcus aureus* (MTCC96) by employing the inhibition zone technique [18]. In this technique, a nutrient agar medium comprising 5 g peptone, 3 g beef extract, 20 g agar-agar, and 5 g NaCl were suspended in 1000 mL distilled water, which was further boiled, allowing all the ingredients to dissolve completely. The prepared agar medium was then poured into the petriplates thereby allowing it to solidify. Solutions of the test compounds in methanol with 500 and 1000 ppm concentrations were prepared. The Whatman No. 1 paper discs with 5 mm diameter, soaked in different solutions of the compounds were dried and then placed in petriplates previously seeded with the test organisms. The petri dishes were then incubated at a temperature of 28 ± 2°C for duration of 24 hrs. DMSO was used as a negative control while a disc of streptomycin was used as a positive control for antibacterial activity. The zone of inhibition thus formed around each disc encompassing the test compounds was measured accurately and used as indices of antimicrobial actions.

*(2) Antifungal Activity*. The efficacy of the synthesized complexes was evaluated against two of the fungal strains *Fusarium oxysporum* and *Aspergillus niger* using the agar plate technique [19]. In the current technique, potato dextrose agar media is prepared consuming 20 g starch, 20 g dextrose, and 20 g agar-agar dissolved in 1000 mL of distilled water. The compounds were directly mixed with the medium in 50, 100, and 200 ppm concentrations dissolved in methanol. The medium was transferred into the petriplates onto which spores of fungi were placed with the help of an inoculum needle. These petriplates were wrapped in the polythene bags containing a few drops of alcohol and were placed in an incubator at 25 ± 2°C. The standard drug Bavistin was used as a positive control for the antifungal activity while DMSO was used as a negative control. The linear growth of the fungus was obtained by measuring the diameter of the fungal colony after four days (96 hrs).

The growth of fungi was analyzed, and the percentage of inhibition was calculated by the following equation:

(1) $$\begin{array}{c} \%\;\mathrm{inhibition}=\frac{C-T}{C}\times 100, \end{array}$$

where *C* and *T* are the diameters of the fungal colony in the control and the test plates, respectively.

*(3) Hepatoprotective Activity*. The hepatoprotective activity was evaluated by carrying out an experiment with the male albino rats weighing about 180–200 g distributed into four groups of 10 rats each. The group I was treated as control, while hepatotoxicity was introduced in the rats belonging to groups II, III, and IV via oral administration of CCl_4_ of about 0.25 mL/100 g body weight. Animals were fed twice in a week for about 4 weeks. Commencing the fifth day, the animals of groups II and III were provided with an oral dose (50 mg/100 g bw for 30 days) of the compounds [Cu(C_6_H_5_N_2_F)_2_Sn_2_(Ph)_4_Cl_2_] and [Cu(C_6_H_5_N_2_F)_2_Sn_2_(CH_3_)_4_Cl_2_], respectively. All the animals were fed on commercial standard pellet diet (Hindustan Lever Ltd., Mumbai) with water ad libitum and were maintained in the animal house at a temperature of 25 ± 2°C under 12 hr light/dark cycle with a relative humidity of about 60 ± 5%.

Further experiment required blood of the animals for which three rats were scarified every week. These blood samples were collected by direct heart puncture into a sterilized dried out centrifuge tube while serum was collected for the evaluation of total bilirubin, protein, and albumin/globulin ratio [20]. It is observed that the toxic effect of CCl_4_ causes harm to the liver resulting in its disfuctioning in the experimental animals, and analogous results are anticipated in the human viral hepatitis model. In CCl_4_-induced toxic hepatitis, a toxic reactive metabolite, trichloromethyl radical was produced by the microsomal oxidase system that binds covalently to the macromolecules of the lipid membranes of the endoplasmic reticulum resulting in peroxidative degradation of lipids. As an outcome, adipose tissue from the fats gets translocated and further accumulates down in the liver. In majority of cases, this toxic chemical is used as a tool to instigate hepatotoxicity in experimental animals.

## 3. Results and Discussion

### 3.1. Chemistry

The resulting unimetallic and heterobimetallic complexes are color solids with sharp melting point. The synthesized complexes are stable at room temperature and sparingly soluble in cold organic solvents like methanol, ethanol, and benzene but completely soluble in hot solvents. The molar conductance measurements of 10^−3^ M solution in DMF indicate that the synthesized complexes are 1 : 2 electrolytes. The conductivity measured for the unimetallic complex have conductance of 232 ohm^−1^·mol^−1^·cm^2^ holding electrolytic character while heterobimetallic complexes exhibit conductance in the range of 20–35 ohm^−1^·mol^−1^·cm^2^ suggesting the nonelectrolytic character of these complexes [21]. The analytical and physical data of the synthesized complexes have been precised in Supplementary Table 1.

#### 3.1.1. Infrared Spectra

The IR spectra of all the newly synthesized complexes were recorded and summarized in Supplementary Table 2. However, a comparative study was made between the spectra of unimetallic and heterobimetallic complexes of Cu(II). The primary amine exhibits a band at higher frequency than that of the corresponding secondary amine. In case of unimetallic complex, a broad and strong band appears for *ν*(N–H) in the range of 3180–3256 cm^−1^, which swipes over to a lower frequency region in case of heterobimetallic complexes approving the formation of bond between metal and nitrogen, while bands due to *δ*(N–H) appear in the region of 1535–1546 cm^−1^ with no apparent change after chelation [22]. Bands at 1643, 1528, and 1453 cm^−1^ appear due to (C=C) stretching in case of aromatic ring, while bands due to (C–H) and (C–N) stretching appear at 3057 and 844 cm^−1^, respectively. Bands of medium intensity appearing in the range of 420–585 cm^−1^ are attributed to (M–N) vibrations [23]. In case of heterobimetallic complexes of copper with titanium and zirconium, the presence of cyclopentadienyl ring is confirmed by the IR bands appearing at 3000 for *ν*(C–H), 1433 for *ν*(C–C), 1030 for *δ*(C–H) in plane, and 812 for *δ*(C–H) out of plane vibrations. In addition, bands due to (Ti–C_5_H_5_) and (Zr–C_5_H_5_) appear at 445 and 442 cm^−1^, respectively. A medium intensity band observed in the far IR region of the metal complexes (460–470) cm^−1^ was assigned to (Cu–N). The far IR spectra show a distinct band at 317 cm^−1^ attributed to (Cu–Cl) band [24], which clearly indicates the presence of chlorine bonded with central metal (i.e., Cu) ion. Hence, the IR spectra support an octahedral geometry in case of heterobimetallic complexes further verified by the ESR spectra.

#### 3.1.2. Electron Spin Resonance Spectra

EPR studies of synthesized complexes were carried out on the X-band at 9.1 GHz under the magnetic field strength 3000 G. It is well known that in the case of tetragonal and square planar complexes, the unpaired electron lies in the *d*_*x*^2^−*y*^2^_ orbital giving ^2^B_1g_ as the ground state with *g*_‖_ > *g*_┴_ [1]. The data obtained in case of unimetallic complex shows *g*_‖_ = 2.09 and *g*_┴_ = 2.05, respectively. Thus, for the copper complexes with *g*_‖_ > *g*_┴_ supports the fact that the ground state of Cu(II) is ^2^B_1g_ with the unpaired electron in the *d*_*x*^2^−*y*^2^_ orbital. The observed *g* values for the synthesized monometallic copper complex lie in the range reported for square planar complexes; thus, the EPR spectral studies strongly support the square planar structure for unimetallic complex. Their square planar geometry has also been verified from electronic spectra. However, in case of heterobimetallic complexes, *g*_┴_ > *g*‖ is observed thereby proposing a distorted octahedral geometry with *d*_*z*^2^_ as the ground state of the system [25]. On this basis, it is decided that chloride ions coordinate with Cu(II) on complexation with organometallic dichlorides in case of heterobimetallic complexes thus achieving an octahedral environment for copper in these complexes.

#### 3.1.3. Electronic Spectra

In the electronic spectrum of unimetallic complex, a strong band is observed at 589 nm, which is due to d-d transitions, and is a characteristic feature of the square planar complexes while a strong band at 297 nm and shoulders arise due to the intraligand transitions [26]. In case of heterobimetallic complexes of copper, two bands are witnessed at 920 nm and 875 nm, respectively. This displacement of d-d bands to lower energies is basically due to the distortion in the octahedral geometry of copper(II) complexes.

#### 3.1.4. Mass Spectroscopy

The FAB mass spectra of the heterobimetallic complex [Cu(C_6_H_5_N_2_F)_2_Sn_2_(Ph)_4_Cl_2_] exhibited molecular ion peaks at *m*/*z* 928, 890, 813, 736, 617, and 344 assigned to [Cu(C_6_H_5_N_2_F)_2_Sn_2_(Ph)_4_Cl_2_]^+^, [Cu(C_6_H_5_N_2_)_2_Sn_2_(Ph)_4_Cl_2_]^+^, [Cu(C_6_H_5_N_2_)_2_Sn_2_(Ph)_3_Cl_2_]^+^, [Cu(C_6_H_5_N_2_)_2_Sn_2_(Ph)_2_Cl_2_]^+^, [Cu(C_6_H_5_N_2_)_2_Sn(Ph)_2_Cl_2_]^+^, and [Cu(C_6_H_5_N_2_)_2_Cl_2_]^+^. The two coordinated chlorides are removed with a mass loss of *m*/*z* = 71 with peak obtained at *m*/*z* = 273 (Supplementary Figure 1).

#### 3.1.5. X-Ray Powder Diffraction Studies

The possible geometry of the finely powdered mononuclear complex [Cu(C_6_H_7_N_2_F)_2_]Cl_2_ has been inferred on the basis of X-ray powder diffraction studies (Supplementary Figure 2). The interplanar spacing values (“*d*” in Å) of this complex have been measured from the diffractogram and the Miller indices *h*, *k*, and *l* have been assigned to each *d* value as reported in Supplementary Table 3. The results display that compounds belong to “orthorhombic” crystal system with the unit cell parameters *a* = 9.480, *b* = 13.416, *c* = 21.193, and *α* = *β* = *γ* = 90°.

**Figure 1:**
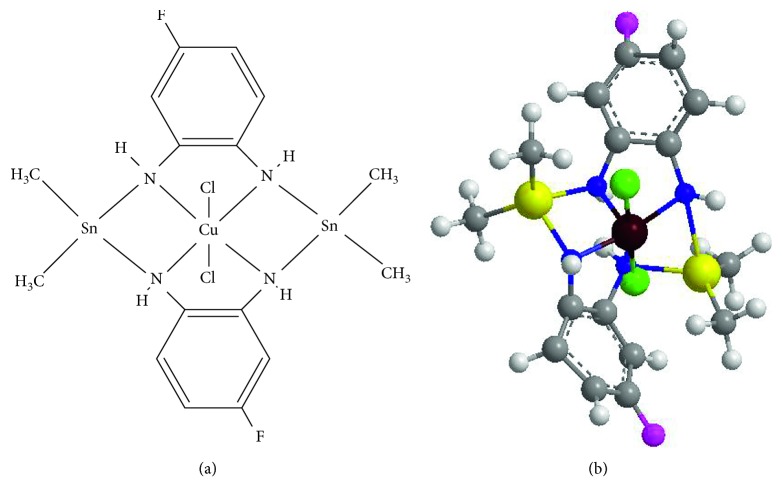
Proposed structure for heterobimetallic complex [Cu(C_6_H_5_N_2_F)_2_Sn_2_(CH_3_)_4_Cl_2_].

From the data obtained using various physicochemical techniques such as IR, ESR, mass, and X-ray powder diffraction studies, the structure proposed for heterobimetallic complex is depicted in Figure 1.

### 3.2. Biological Assay

#### 3.2.1. Antimicrobial Activity

The data obtained after evaluating the synthesized complexes against two bacterial (*E. coli* and *S. aureus*) strains and two fungal (*F. oxysporum* and *A. niger*) strains evidently divulges the amplified activity of heterobimetallic complexes in comparison to unimetallic complexes of copper as summarized in Tables 1 and 2. This can be explained on the basis of Tweedy's chelation theory [27] according to which, the polarity of metal ion reduces due to chelation owing to the partial sharing of its positive charge with the donor groups and possible *π*-electron-delocalization over the whole chelation ring, which intensifies the lipophilic character of the metal complex, consecutively approving its penetration through the lipid layers of the organism cell membrane, resulting in reduction of the normal cell process along with deactivation of numerous cellular enzymes that play dictatorial roles in the various metabolic pathways of these microorganisms. Hence, it is clear that amid all the synthesized complexes, unimetallic complexes of Cu(II) are lesser active, that is, having less significant antimicrobial efficacy as compared to heterobimetallic complexes where the synergistic effect of two metals displays remarkable biological activity.

**Figure 2:**
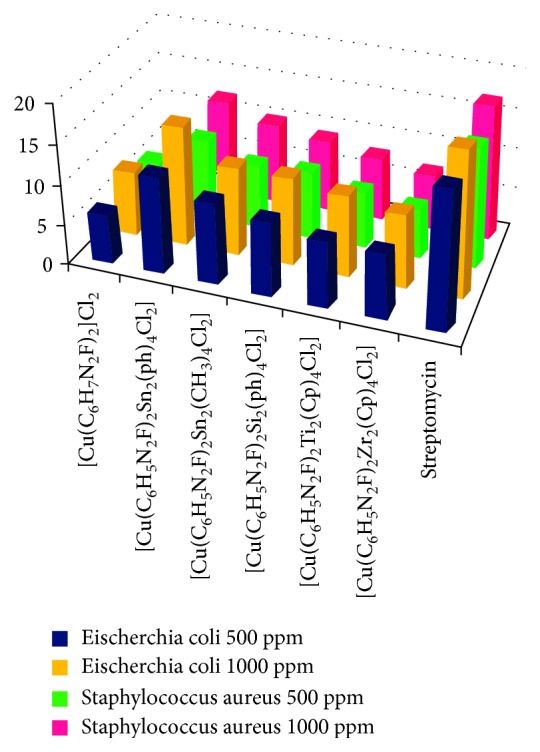
Bactericidal activity of the synthesized Cu(II) complexes.

**Figure 3:**
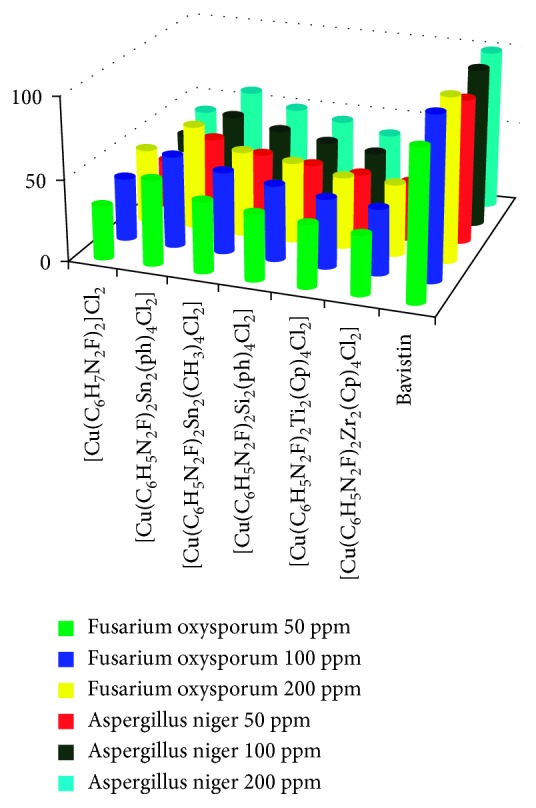
Fungicidal activity of the synthesized Cu(II) complexes.

In the present series, heterobimetallic complex [Cu(C_6_H_5_N_2_F)_2_Sn_2_(Ph)_4_Cl_2_] exhibits the unsurpassed antifungal and antibacterial activity thus presenting enriched antimicrobial efficacy as equated to the unimetallic complex of Cu(II) (Figures 2 and 3).

#### 3.2.2. Appraisal of Hepatoprotective Activity

There are number of biochemical parameters used for the evaluation of hepatoprotective activity in carbon tetrachloride-induced toxicity which comprises bilirubin, protein, albumin, globulin, aspartate aminotransaminase, alkaline phosphatase, and alanine aminotransaminase (Table 3). The prominent inferences drawn from the experiment are as follows:

1. The present study revealed a substantial escalation in the levels of bilirubin, globulin, aspartate aminotransaminase, alanine aminotransaminase, and alkaline phosphatase in the blood samples of the group bearing animals treated with CCl_4_. The rise in the levels of these biochemical parameters is a clear indication of cellular leakage with a loss of functional integrity of the cell membrane.
2. The intensity of jaundice is confirmed from the total amount of bilirubin present with a normal range of 0.2 to 1 mg/100 mL of serum. However, it is observed that hyperbilirubinemia occurs more in case of hepatitis patients with the excretion of bilirubin present in the liver into the canaliculi and then regurgitated into the blood stream. The group of animals treated with compounds [Cu(C_6_H_5_N_2_F)_2_Sn_2_(Ph)_4_Cl_2_] and [Cu(C_6_H_5_N_2_F)_2_Sn_2_(CH_3_)_4_Cl_2_] exhibits reduced levels of total bilirubin in the blood samples.
3. A number of serum proteins are being synthesized by the liver which plays an imperative role in the diagnosis of jaundice. Further, there is a correlation between the degree of serum hypoalbuminemia and hyperglobulinemia [28]. Normally, the ratio of albumin and globulin lies in the range of 2 : 1. However, in case of CCl_4_-treated animals, there is a significant reduction in the levels of total protein and albumin with the increase in globulin level. Even with the toxic effect of CCl_4_, the complexes [Cu(C_6_H_5_N_2_F)_2_Sn_2_(Ph)_4_Cl_2_] and [Cu(C_6_H_5_N_2_F)_2_Sn_2_(CH_3_)_4_Cl_2_] were highly proficient in refurbishing the reduced and increased levels of serum total protein and globulin, respectively.

The percentage of serum protein level restored was found to be more in case of heterobimetallic complex [Cu(C_6_H_5_N_2_F)_2_Sn_2_(Ph)_4_Cl_2_].

**Figure 4:**
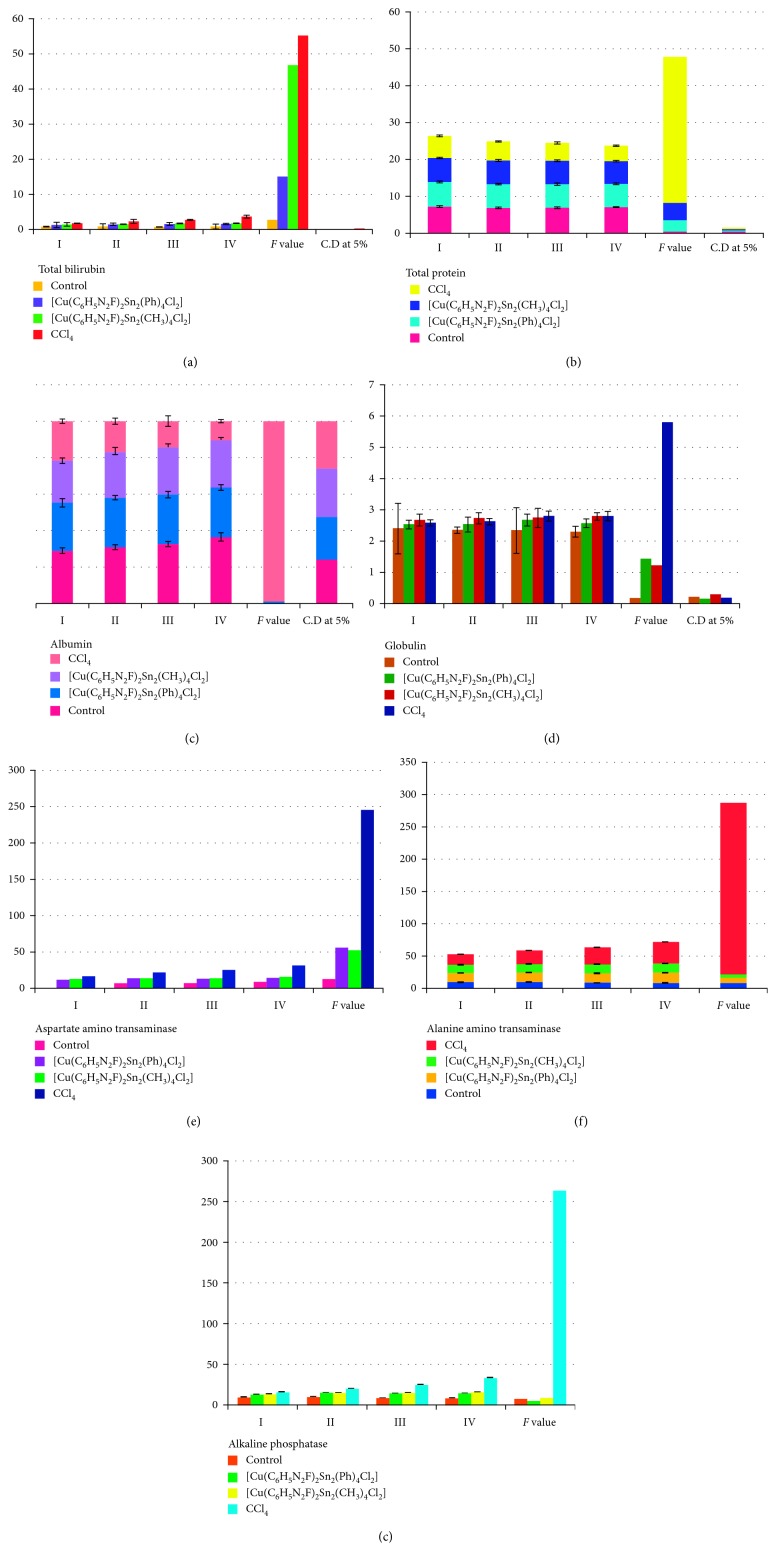
Graphical representation of various biochemical parameters: (a) total bilirubin, (b) total protein, (c) albumin, (d) globulin, (e) aspartate aminotransaminase, (f) alanine aminotransaminase, and (g) alkaline phosphatase as compared to the control.

(4) The normal values of aspartate aminotransaminase (ALT), alanine aminotransaminase (AST), and alkaline phosphatase (ALP) ranges from 5 to 20, 5 to 15, and 7 to 9 IU/mg of protein, respectively. These enzymes present in the serum are supportive in the diagnosis of hepatitis disease while an upsurge in the concentration of these enzymes is detected with the damage in the liver tissue, which is ostensibly due to the release of these enzymes from the damaged cells. In acute hepatic necrosis, the level of AST and ALT are estimated to rise by 2 to 20 folds over that of controls while on the other hand, in case of obstructive and posthepatic jaundice, escalation of ALP was more. In the current research, the group of animals treated with CCl_4_ exhibits remarkable increase in the activities of these enzymes parallelly from 1st to 4th week of treatment. While complexes [Cu(C_6_H_5_N_2_F)_2_Sn_2_(Ph)_4_Cl_2_] and [Cu(C_6_H_5_N_2_F)_2_Sn_2_(CH_3_)_4_Cl_2_] prohibited the increase in the levels of these enzymes, presenting the pattern of recovery from the toxic effect (Figure 4).

## 4. Conclusion

The present study unveiled the biological potency of heterobimetallic complexes of copper synthesized using group 4 and 14 organometallic dichlorides. All these synthesized unimetallic and heterobimetallic complexes have been structurally characterized using varied techniques such as IR, ESR, electronic spectra, mass spectra, and X-ray diffraction studies. The spectroscopic data revealed square planar and distorted octahedral geometry for mononuclear and heterobimetallic complexes, respectively. These heterobimetallic complexes possess high biological activity emerging from the synergistic effect arising due to the two different metal centers seized together in close proximity. The antimicrobial-screening data of these complexes indicate that heterobimetallic complexes are more active against these microbes as compared to the unimetallic complexes with heterobimetallic complex of copper with tin proving out to be the best. The heterobimetallic complexes with tin were also evaluated for the hepatoprotective activity in the male albino rats taking into account different biochemical parameters.

## Figures and Tables

**Table 1:** Bacterial screening data of the synthesized Cu(II) complexes (percent growth inhibition after 24 hrs at 28 ± 2°C).

Complex	*Escherichia coli*		*Staphylococcus aureus*	
	500 ppm	1000 ppm	500 ppm	1000 ppm
[Cu(C_6_H_7_N_2_F)_2_]Cl_2_	6	8	6	7
[Cu(C_6_H_5_N_2_F)_2_Sn_2_(Ph)_4_Cl_2_]	12	15	10	12
[Cu(C_6_H_5_N_2_F)_2_Sn_2_(CH_3_)_4_Cl_2_]	10	11	8	10
[Cu(C_6_H_5_N_2_F)_2_Si_2_(Ph)_4_Cl_2_]	9	11	8	9
[Cu(C_6_H_5_N_2_F)_2_Ti_2_(Cp)_4_Cl_2_]	8	10	7	8
[Cu(C_6_H_5_N_2_F)_2_Zr_2_(Cp)_4_Cl_2_]	8	9	6	7
Streptomycin	17	18	15	17

**Table 2:** Fungicidal screening data of the synthesized Cu(II) complexes (percent growth inhibition after 96 hrs at 25 ± 2°C, conc. in ppm).

Complex	*Fusarium oxysporum*			*Aspergillus niger*		
	50 ppm	100 ppm	200 ppm	50 ppm	100 ppm	200 ppm
[Cu(C_6_H_7_N_2_F)_2_]Cl_2_	32	38	46	28	35	41
[Cu(C_6_H_5_N_2_F)_2_Sn_2_(Ph)_4_Cl_2_]	52	56	64	46	51	58
[Cu(C_6_H_5_N_2_F)_2_Sn_2_(CH_3_)_4_Cl_2_]	43	50	52	40	45	49
[Cu(C_6_H_5_N_2_F)_2_Si_2_(Ph)_4_Cl_2_]	40	46	49	37	41	45
[Cu(C_6_H_5_N_2_F)_2_Ti_2_(Cp)_4_Cl_2_]	38	42	44	35	38	40
[Cu(C_6_H_5_N_2_F)_2_Zr_2_(Cp)_4_Cl_2_]	36	40	43	33	35	37
Bavistin	91	100	100	89	98	100

**Table 3:** Hepatoprotective activity of heterobimetallic complexes of Cu(II).

Group of animals	Time in weeks				*F* value	C.D at 5%
	I	II	III	IV		
*Total bilirubin*						
Control	0.85 ± 0.10	0.86 ± 0.79	0.73 ± 0.06	0.86 ± 0.69	2.74	0.018
[Cu(C_6_H_5_N_2_F)_2_Sn_2_(Ph)_4_Cl_2_]	1.32 ± 0.75	1.48 ± 0.38	1.56 ± 0.45	1.61 ± 0.18	15.1	0.071
[Cu(C_6_H_5_N_2_F)_2_Sn_2_(CH_3_)_4_Cl_2_]	1.46 ± 0.50	1.52 ± 0.08	1.75 ± 0.10	1.80 ± 0.08	46.8	0.084
CCl_4_	1.80 ± 0.05	2.30 ± 0.60	2.75 ± 0.15	3.70 ± 0.41	55.2	0.315

*Total protein*						
Control	7.24 ± 0.23	6.87 ± 0.22	6.90 ± 0.25	7.08 ± 0.10	0.451	0.442
[Cu(C_6_H_5_N_2_F)_2_Sn_2_(Ph)_4_Cl_2_]	6.67 ± 0.25	6.44 ± 0.20	6.40 ± 0.30	6.30 ± 0.24	3.10	0.348
[Cu(C_6_H_5_N_2_F)_2_Sn_2_(CH_3_)_4_Cl_2_]	6.50 ± 0.16	6.39 ± 0.27	6.36 ± 0.25	6.15 ± 0.22	4.69	0.342
CCl_4_	5.98 ± 0.24	5.20 ± 0.17	4.82 ± 0.29	4.19 ± 0.17	39.6	0.350

*Albumin*						
Control	4.68 ± 0.25	4.50 ± 0.20	4.50 ± 0.20	4.78 ± 0.30	0.45	0.376
[Cu(C_6_H_5_N_2_F)_2_Sn_2_(Ph)_4_Cl_2_]	4.23 ± 0.37	3.97 ± 0.17	3.75 ± 0.25	3.55 ± 0.20	4.49	0.366
[Cu(C_6_H_5_N_2_F)_2_Sn_2_(CH_3_)_4_Cl_2_]	3.72 ± 0.24	3.62 ± 0.40	3.60 ± 0.25	3.42 ± 0.17	1.61	0.416
CCl_4_	3.50 ± 0.20	2.50 ± 0.25	1.97 ± 0.40	1.35 ± 0.12	414.9	0.405

*Globulin*						
Control	2.40 ± 0.81	2.35 ± 0.10	2.34 ± 0.73	2.30 ± 0.17	0.18	0.207
[Cu(C_6_H_5_N_2_F)_2_Sn_2_(Ph)_4_Cl_2_]	2.53 ± 0.14	2.53 ± 0.24	2.67 ± 0.19	2.57 ± 0.14	1.44	0.150
[Cu(C_6_H_5_N_2_F)_2_Sn_2_(CH_3_)_4_Cl_2_]	2.67 ± 0.19	2.73 ± 0.18	2.74 ± 0.31	2.79 ± 0.12	1.23	0.295
CCl_4_	2.58 ± 0.10	2.62 ± 0.10	2.80 ± 0.16	2.80 ± 0.15	5.80	0.183

*Aspartate aminotransaminase*						
Control	0.45 ± 0.10	7.50 ± 0.24	7.78 ± 0.50	9.20 ± 0.20	12.9	0.598
[Cu(C_6_H_5_N_2_F)_2_Sn_2_(Ph)_4_Cl_2_]	11.55 ± 0.40	14.25 ± 0.35	13.90 ± 0.55	15.10 ± 0.35	56.10	0.580
[Cu(C_6_H_5_N_2_F)_2_Sn_2_(CH_3_)_4_Cl_2_]	13.0 ± 0.40	14.15 ± 0.24	14.32 ± 0.25	16.40 ± 0.65	52.61	0.602
CCl_4_	16.80 ± 0.40	22.20 ± 0.80	25.76 ± 0.70	32.20 ± 1.0	245.7	1.160

*Alanine aminotransaminase*						
Control	9.65 ± 0.65	9.85 ± 0.51	8.65 ± 0.20	8.40 ± 0.65	7.90	6.729
[Cu(C_6_H_5_N_2_F)_2_Sn_2_(Ph)_4_Cl_2_]	14.32 ± 0.27	14.82 ± 0.63	14.82 ± 0.79	15.93 ± 0.45	8.95	0.896
[Cu(C_6_H_5_N_2_F)_2_Sn_2_(CH_3_)_4_Cl_2_]	12.73 ± 0.62	13.24 ± 0.66	14.04 ± 0.52	14.42 ± 0.27	5.18	0.990
CCl_4_	16.10 ± 0.30	20.90 ± 0.24	26.00 ± 0.43	33.40 ± 1.5	265.0	1.376

*Alkaline phosphatase*						
Control	9.67 ± 0.50	9.85 ± 0.50	8.65 ± 0.20	8.45 ± 0.64	7.75	0.728
[Cu(C_6_H_5_N_2_F)_2_Sn_2_(Ph)_4_Cl_2_]	12.85 ± 0.50	14.83 ± 0.52	14.06 ± 0.52	14.48 ± 0.30	5.18	0.985
[Cu(C_6_H_5_N_2_F)_2_Sn_2_(CH_3_)_4_Cl_2_]	13.40 ± 0.60	14.77 ± 0.43	14.87 ± 0.43	15.61 ± 0.42	8.95	0.886
CCl_4_	16.10 ± 0.34	20.24 ± 0.24	25.00 ± 0.44	33.60 ± 1.46	263.5	1.278

## Supplementary Materials

Supplementary Materials Graphical abstract, Tables 1–3, and Figures 1 and 2.
