# Supplementary material for: Synthesis, Characterization, and Biological Evaluation of Unimetallic and Heterobimetallic Complexes of Bivalent Copper
Source: Bioinorg Chem Appl. 2018 Apr 12;2018:2467463. doi: 10.1155/2018/2467463 (PMC5925014; doi:10.1155/2018/2467463)
Supplement: Supplementary Materials — Graphical abstract, Tables 1–3, and Figures 1 and 2. [file 2467463.f1.docx]

**Graphical abstract:**

**
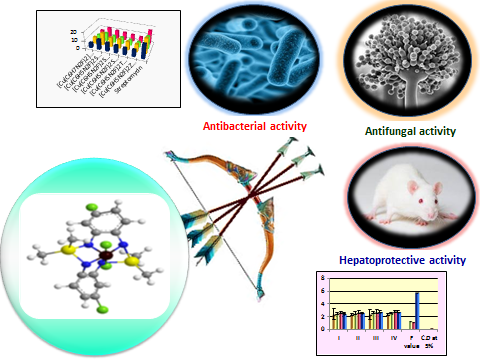
**

| **Complex** | **Color &**  **M.P**  **(˚C)** | **Analysis: found (calc.) (%)** | | | | | | **Mol. Wt.**  **Found (calc.)** |
| --- | --- | --- | --- | --- | --- | --- | --- | --- |
|  |  | **C** | **H** | **N** | **Cl** | **Cu** | **M'** |  |
| [Cu(C_6_H_7_N_2_F)_2_]Cl_2_ | 229  Brown | 36.04  (37.27) | 3.32  (3.65) | 13.85  (14.49) | 18.36  (18.34) | 16.03  (16.43) | - | 364.45  (386.72) |
| [Cu(C_6_H_5_N_2_F)_2_Sn_2_(Ph)_4_Cl_2_] | 245  Dark brown | 44.28  (46.57) | 2.89  (3.26) | 5.76  (6.03) | 6.98  (7.64) | 6.21  (6.84) | 24.12  (25.57) | 910.52  (928.52) |
| [Cu(C_6_H_5_N_2_F)_2_Sn_2_(CH_3_)_4_Cl_2_] | 238  Dark brown | 27.01  (28.25) | 2.84  (3.26) | 7.81  (8.24) | 10.05  (10.42) | 8.93  (9.34) | 33.71  (34.90) | 665.61  (680.24) |
| [Cu(C_6_H_5_N_2_F)_2_Si_2_(Ph)_4_Cl_2_] | 232  Light brown | 56.14  (57.86) | 3.86  (4.05) | 6.91  (7.50) | 8.83  (9.49) | 7.89  (8.50) | 7.06  (7.52) | 724.58  (742.27) |
| [Cu(C_6_H_5_N_2_F)_2_Ti_2_(Cp)_4_Cl_2_] | 241  Reddish brown | 51.10  (52.02) | 3.89  (4.09) | 6.97  (7.58) | 9.01  (9.60) | 8.00  (8.60) | 11.83  (12.96) | 715.79  (738.79) |
| [Cu(C_6_H_5_N_2_F)_2_Zr_2_(Cp)_4_Cl_2_] | 249  Shiny dark brown | 45.23  (46.56) | 3.21  (3.66) | 6.21  (6.79) | 7.98  (8.59) | 6.99  (7.70) | 21.54  (22.10) | 805.2  (825.5) |

TABLE 1: The physical properties and analytical data of synthesized complexes of Cu(II).

TABLE 2: IR spectral data (in cm^-1^) of the synthesized unimetallic and heterobimetallic complexes.

| **Complex** | **ν(N-H)** | **δ(N-H)** | **ν(M-N)** | **ν(Cu-N)** | **ν(Cu-Cl)** |
| --- | --- | --- | --- | --- | --- |
| [Cu(C_6_H_7_N_2_F)_2_]Cl_2_ | 3256 | 1546 | - | 467 | - |
| [Cu(C_6_H_5_N_2_F)_2_Sn_2_(Ph)_4_Cl_2_] | 3225 | 1538 | 424 | 465 | 317 |
| [Cu(C_6_H_5_N_2_F)_2_Sn_2_(CH_3_)_4_Cl_2_] | 3190 | 1538 | 420 | 463 | 317 |
| [Cu(C_6_H_5_N_2_F)_2_Si_2_(Ph)_4_Cl_2_] | 3186 | 1536 | 580 | 463 | 317 |
| [Cu(C_6_H_5_N_2_F)_2_Ti_2_(Cp)_4_Cl_2_] | 3180 | 1538 | 530 | 464 | 317 |
| [Cu(C_6_H_5_N_2_F)_2_Zr_2_(Cp)_4_Cl_2_] | 3198 | 1538 | 535 | 468 | 317 |

TABLE 3: X-ray powder diffraction data for the compound [Cu(C_6_H_7_N_2_F)_2_)]Cl_2._

| **Peak No.** | **2θ (deg.)**  **(obs.)** | **h** | **k** | **l** | **d-spacing**  **(obs.)** |
| --- | --- | --- | --- | --- | --- |
| 1. | 9.334 | 1 | 0 | 0 | 9.465 |
| 2. | 11.451 | 1 | 1 | 0 | 7.720 |
| 3. | 16.934 | 0 | 0 | 4 | 5.232 |
| 4. | 19.097 | 2 | 0 | 1 | 4.641 |
| 5. | 25.525 | 2 | 0 | 3 | 3.939 |
| 7. | 26.910 | 0 | 4 | 1 | 3.310 |
| 8. | 28.078 | 2 | 3 | 0 | 3.169 |
| 9. | 29.207 | 3 | 1 | 1 | 3.056 |
| 10. | 31.190 | 3 | 2 | 0 | 2.860 |

a =9.480, b =13.416, c =21.193; α=β=γ=90° (orthorhombic system).


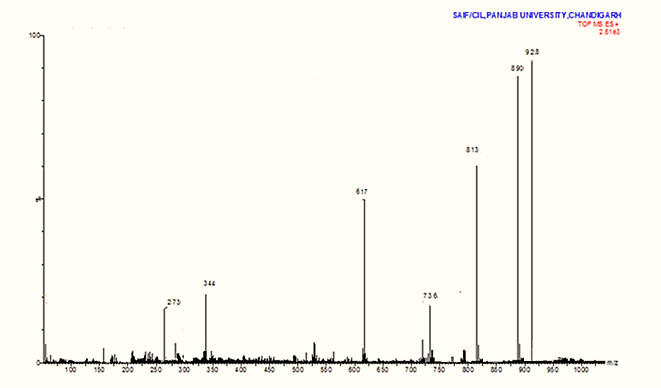


FIGURE 1: Mass spectra of heterobimetallic complex [Cu(C_6_H_5_N_2_F)_2_Sn_2_(Ph)_4_Cl_2_)].


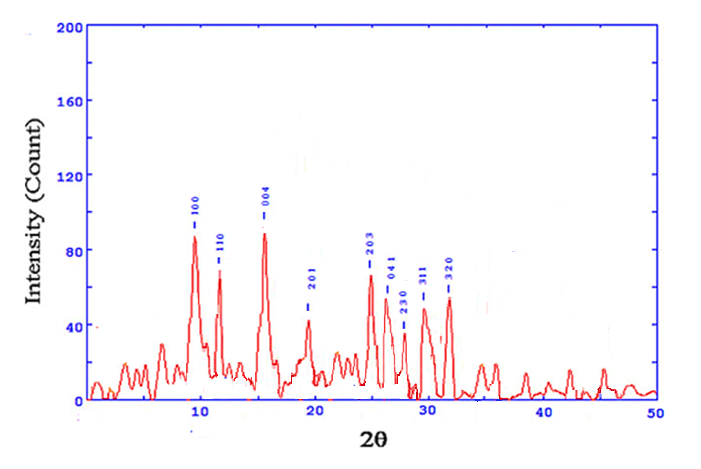


FIGURE 2: X-ray diffraction pattern of compound [Cu(C_6_H_7_N_2_F)_2_)]Cl_2._
